# Systematic Review of Chemical Constituents in the Genus *Lycium* (Solanaceae)

**DOI:** 10.3390/molecules22060911

**Published:** 2017-06-08

**Authors:** Dan Qian, Yaxing Zhao, Guang Yang, Luqi Huang

**Affiliations:** 1 Experimental Research Center, China Academy of Chinese Medical Sciences, Beijing 100700, China; qiandan2104@163.com; 2 The State Key Laboratory Breeding Base of Dao-di Herbs, National Resource Center for Chinese Materia Medica, China Academy of Chinese Medical Sciences, Beijing 100700, China; hbykdxyg2008@163.com; 3 School of Pharmaceutical Science and Technology, Tianjin University, Tianjin 300072, China; zhaoyaxing2013@163.com

**Keywords:** *Lycium* genus, chemical constituents, goji berry, Lycii cortex

## Abstract

The *Lycium* genus is widely used as a traditional Chinese medicine and functional food. Many of the chemical constituents of the genus *Lycium* were reported previously. In this review, in addition to the polysaccharides, we have enumerated 355 chemical constituents and nutrients, including 22 glycerogalactolipids, 29 phenylpropanoids, 10 coumarins, 13 lignans, 32 flavonoids, 37 amides, 72 alkaloids, four anthraquinones, 32 organic acids, 39 terpenoids, 57 sterols, steroids, and their derivatives, five peptides and three other constituents. This comprehensive study could lay the foundation for further research on the *Lycium* genus.

## 1. Introduction

*Lycium* is one of the genera in the Solanaceae family, comprising 80 species, seven of which are found in China [1]. These species are all deciduous shrubbery, possessing a highly similar morphology and structure. The *Lycium* genus has been an important source of medicines and nutrient supplements for thousands of years in Southeast Asia, especially in China. Two species in particular, *Lycium barbarum* and *Lycium chinense*, have been widely used as traditional Chinese medicinal herbs for centuries and *L. barbarum* is currently widely cultivated in China.

Goji berries (Chinese name Gouqizi), which are derived from the fruits of *Lycium* Linn, have been used as traditional herbs for a long time in China for their benefits of replenishing vital essence to improve eyesight, nourish the liver and kidneys. Lycii cortex is a “heat cleansing” drug that is derived from the root bark of *L. chinense* and *L. barbarum* [2]. Goji berries and Cortex Lycii have demonstrated good therapeutic effects in some chronic diseases such as hectic fever, night sweats, cough, hemoptysis, and diabetes. Recently, medical research has indicated that these fruits and root bark have many pharmacological functions, such as antiglaucoma, immunoregulatory, antitumor, antioxidant, antiaging, neuroprotective, and blood sugar level reducing activities [3,4,5,6,7,8,9,10].

Traditionally, the berry and root bark available have been used as medicinal sources, as well as important components in some traditional Chinese patent medicines. They are not only famous medical herbs, but are also functional foods widely consumed in health-preserving cuisines, i.e., soups, congee, herbal tea, etc. People also eat the fresh leaves as vegetables. In particular, goji berries have become increasingly popular for improving overall well-being and as an anti-aging remedy. There are many goji derived-products on health food market, such as dried fruits, juice, goji wine and goji yoghurt. Many research papers were published focused on the phytochemical fingerprinting and antioxidant activity of these products [11,12,13,14].

Two valuable medicinal herbs, namely *L. barbarum* and *L. chinense*, have received remarkable attention due to their effective clinical therapy, especially in the anti-aging category. In addition, there are increasing numbers of publications about several other *Lycium* plants, i.e., *Lycium ruthenicum* [15,16]. Many researchers have focused great attention on the *Lycium* genus in recent years, and many chemical components from this genus have been isolated. Therefore, a comprehensive and systematic review on the chemical constituents of the *Lycium* genus is much needed.

Most of the published reviews not only covered chemical composition, but also summarized the pharmacology, clinical studies, safety, toxicology and adverse actions of *L. barbarum* or *L. chinense* [17,18,19]. The aim of this review was to focus on chemical constituents in different parts of plants from different species in *Lycium* genus, especially small molecular compounds with updated research reports. This paper comprehensively summarizes the reports of constituents from the genus *Lycium*. Up to 2016, at least 355 constituents were reported from different species in the *Lycium* genus and different parts (fruits, root bark, leaves, seeds, and flowers) of the plant. This review describes the advances in the phytochemistry of the genus *Lycium* from 1975 to 2016, based on the 142 cited references. The reported constituents can be classified as glycerogalactolipids, phenylpropanoids, coumarins, lignans, flavonoids, amides, alkaloids, anthraquinones, organic acids, terpenoids, sterols, steroids, peptides, and other constituents. The aim of this review is to illustrate the recent advances in the characterization of the *Lycium* genus. The results, based on these phytochemical studies, could lay a solid foundation for better understanding of pharmacological activities of *Lycium* and quality assessment.

## 2. Constituents

**Figure 1:**
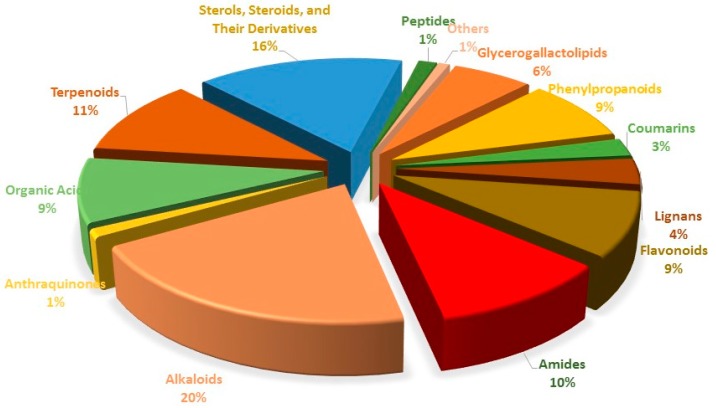
Different subtype comparison of the 355 constituents reported from *Lycium* genus.

Until now, other than polysaccharides, more than 355 compounds have been isolated and identified from the *Lycium* genus. The small molecules can be assigned to various classes of glycerogalactolipids, phenylpropanoids, coumarins, lignans, flavonoids, amides, alkaloids, anthraquinones, organic acids, terpenoids, sterols, steroids and their derivatives, and peptides. Beyond that, other groups of compounds have also been reported. The proportion of different compounds of the *Lycium* genus is show in Figure 1. Their structures are shown below, and their names and corresponding plant sources are included in this paper.

### 2.1. Macromolecules in the Lycium Genus

#### Polysaccharides

Polysaccharides are the most important group of substances in the goji berry, which are estimated to comprise 5–8% of the dried fruits [20], 1.02–2.48% of the raw material [21,22,23]. More than 40 polysaccharides, with a molecular weight range of 8–241 kDa, were isolated from the fruit of *L. barbarum, L. chinense* and *L. ruthenicum.* Two, LRLP4-A and LBLP5-A, were isolated from the leaves of *L. ruthenicum*. The polysaccharides share a glycan-O-Ser glycopeptide structure and contain galacturonic acid, 18 amino acids, and nine monosaccharides, namely, xylose (Xyl), glucose (Glc), arabinose (Ara), rhamnose (Rha), mannose (Man), galactose (Gal), fucose (Fuc), galacturonic acid (GalA), glucuronic acid (GlcA) [24]. The molar ratios of the polysaccharides are shown in Table 1. The polysaccharides can be isolated and purified by water extract alcohol precipitation, DEAE ion-exchange cellulose, gel-permeation chromatography, high performance liquid chromatography (HPLC). Sevage method and organic reagents were used to remove proteins, pigments and other impurities. The structural composition of a LBP can be studied by SDS-PAGE gel electrophoresis, high perfomance size exclusion chromatography (HPSEC), gas-chromatographic–mass-spectrometry (GC-MS), nucleic magnetic resonance (NMR), and matrix-assisted laser desorption ionization-time of flight-mass spectrometry (MALDI-Tof-MS) [18,21,25].

### 2.2. Small Molecule Substances

#### 2.2.1. Glycerogalactolipids **1**–**22**

**Figure 2:**
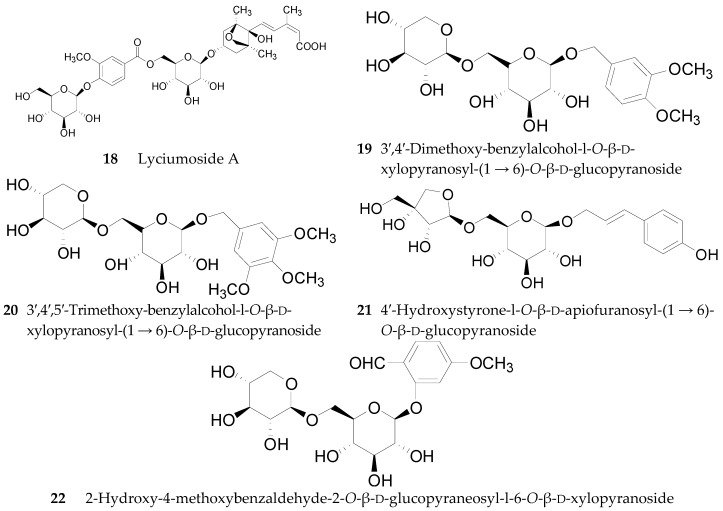
Chemical structures of compounds **18**–**22**.

At present, 17 compounds of this type, a series of glycerogalactolipids **1**–**17**, listed in Table 2, have been isolated and identified. Compounds **1**–**15** have been isolated and identified from the fruits of *L. barbarum* [52], whereas **16** and **17** were isolated from the fruits of *L. chinense* [53]. Compounds **18**–**22**, illustrated in Figure 2, were isolated from the root bark of *L. chinense* [54,55].

#### 2.2.2. Phenylpropanoids **23**–**51**

**Figure 3:**
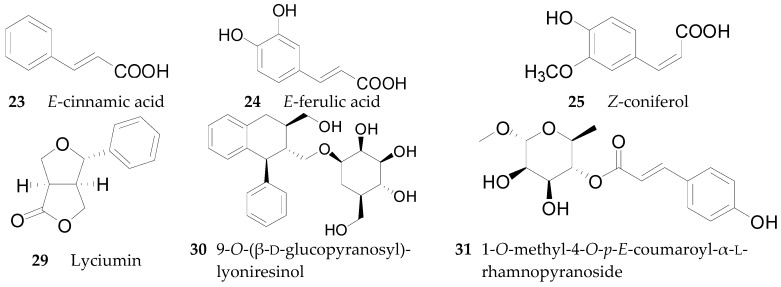
Chemical structures of compounds **23**–**25**, **29**–**31**.

Four phenylpropanoids **23**–**26**, namely *E*-cinnamic acid (**23**), *E*-ferulic acid (**24**), *E*-coniferol (**25**) and isoscopoletin (**26**) are obtained from wolfberries [56,57,58]. Four phenylpropanoids, namely scopolin (**27**), fabiatrin (**28**), lyciumin (**29**), and 9-*O*-(β-d-glucopyranosyl)lyoniresinol (**30**) are obtained from the root bark of *L. chinense* [59,60,61]. 1-*O*-Methyl-4-*O*-*p*-*E*-coumaroyl-α-l-rhamnopyranoside (**31**) is obtained from the fruits of *L. ruthenicum* [62]. The chemical structures of compounds **23**–**33** are listed in Table 3 and Figure 3. In 2016, 11 phenylpropanoids **32**–**42** were isolated for the first time by Zhou et al. from *Lycium* [56], including 1-*O*-*E*-feruloyl-6-*O*-β-d-xylopyranosyl-β-d-glucopyranoside (**32**), 6-*O*-*E*-feruloyl-2-*O*-β-d-glucopyranosyl-α-d-glucopyranoside (**33**), 1-*O*-*E*-feruloyl-β-d-glucopyranoside (**34**), ethyl-4-*O*-β-d-glucopyranosyl-*E*-ferulate (**35**), ethyl *E*-ferulate (**36**), *E*-sinapinic acid (**37**), syringenin (**38**), *Z*-ferulic acid (**39**), phloretic acid (**40**), dihydroferulic acid (**41**), and ethyl dihydroferulate (**42**), along with the nine new lycibarbarphenylpropanoids A–I (compounds **43**–**51**) listed in Table 4.

#### 2.2.3. Coumarins **52**–**61**

**Figure 4:**
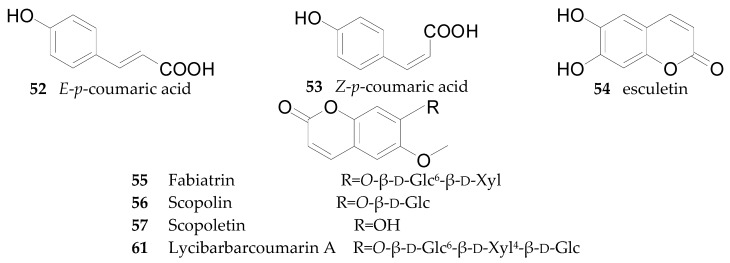
Chemical structures of compounds **52**–**57**, **61**.

Nine coumarins, namely *E*-*p*-coumaric acid (**52**), *Z*-*p*-coumaric acid (**53**), esculetin (**54**), fabiatrin (**55**), scopolin (**56**), and scopoletin (**57**), have been reported, and three new coumarins, 6-*O*-*E*-*p*-coumaroyl-2-*O*-β-d-glucopyranosyl-α-d-glucopyranoside (**58**), ethyl-4-*O*-β-d-glucopyranosyl-*E*-*p*-coumarate (**59**), ethyl *E*-*p*-coumarate (**60**) and lycibarbarcoumarin A (**61**), have been obtained from the fruits of *L. barbarum* in 2016 [56]. Compounds **55** and **56** were isolated from the root bark and fruits of *L. chinense* [61], while **52**−**54** and **57** were isolated from the fruits of *L. barbarum* [63]. The chemical structures of these coumarins are listed in Figure 4 and Table 5.

#### 2.2.4. Lignans **62**–**74**

**Figure 5:**
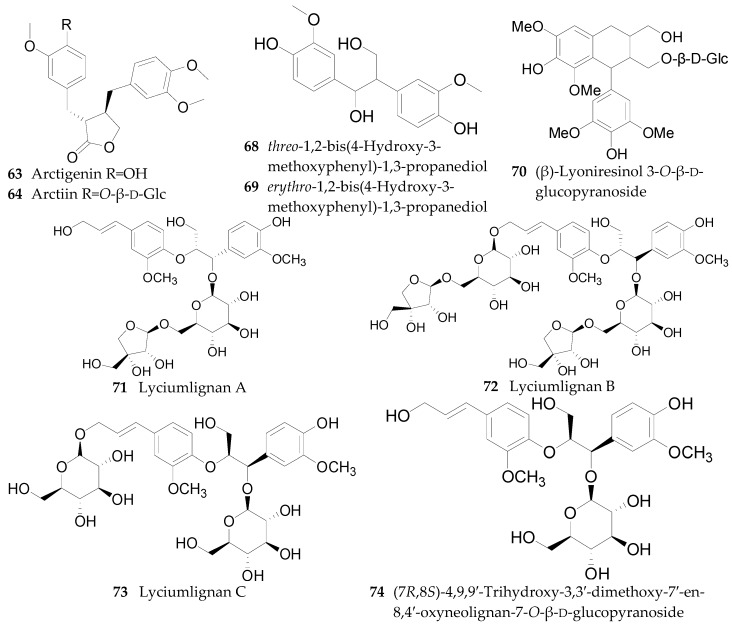
Chemical structures of compounds **63**–**64** and **68**–**74**.

Eight lignans, including pinoresinol (**62**), arctigenin (**63**), arctiin (**64**), medioresinol (**65**), syringaresinol (**66**), 4-*O*-(β-d-glucopyranosyl)syringaresinol (**67**), *threo*-1,2-bis(4-hydroxy-3-methoxy-phenyl)-1,3-propanediol (**68**), and *erythro*-1,2-bis(4-hydroxy-3-methoxyphenyl)-1,3-propanediol (**69**), have been isolated from the fruits of *L. barbarum* [56]. (β)-Lyoniresinol 3-*O*-β-d-glucopyranoside (**70**), lyciumlignan A (**71**), lyciumlignan B (**72**), lyciumlignan C (**73**), and (7*R*,8*S*)-4,9,9′-trihydroxy-3,3′-dimethoxy-7′-en-8,4′-oxyneolignan-7-*O*-β-d-glucopyranoside (**74**) were obtained from the root bark of *L. chinense* [54,60,64]. Among them, **65**–**70** were first isolated from the fruits of *L. barbarum* in 2016 [56]. The chemical structures of these lignans are listed in Figure 5 and Table 6.

#### 2.2.5. Flavonoids **75**–**106**

**Figure 6:**
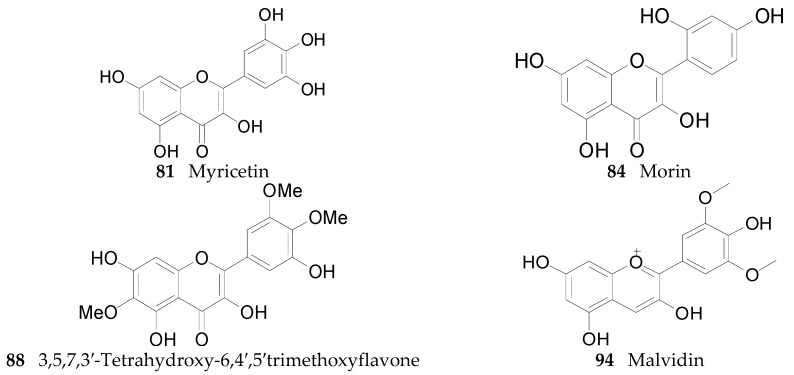
Chemical structures of compounds **81**, **84**, **88** and **94**.

**Figure 7:**
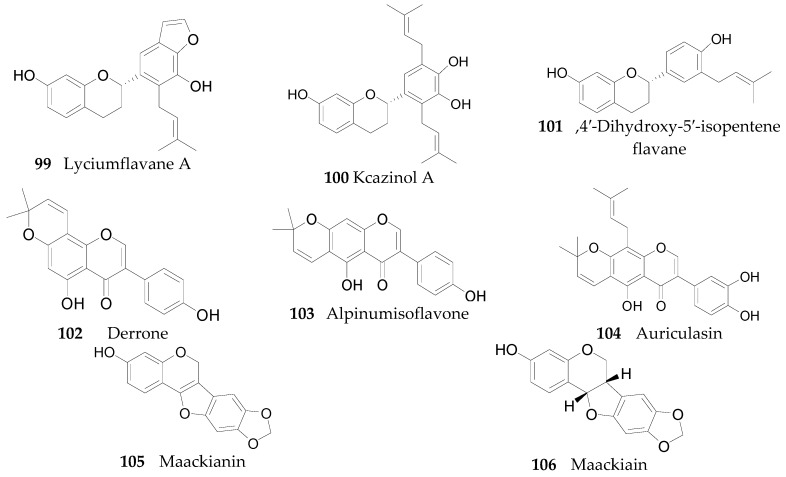
Chemical structures of compounds **99**–**106**.

Twenty-seven flavonoids **75**–**101** have been reported from the genus *Lycium*, are listed in Table 7 and Table 8 and Figure 6 and Figure 7. Compound **75** was isolated from the flowers of *L. barbarum* [58], while **76**–**83** were identified from the fruits of *L. barbarum* [62,65,66,67,68,69]. Compound **84** was isolated from the fruits of *L. chinense* [70], whereas **85**–**91** were isolated from the leaves of *L. chinense* [62,66,68,71]. Compound **92** and **93** were isolated from the leaves of *L. halimifolium* [72]. Compounds **94**–**98** were isolated from the fruits of *L. ruthenicum* [16,62]. Compounds **99**–**101** were isolated from the root bark of *L. chinense* [54,73,74]. Additionally, Zhou et al. isolated five isoflavonoids, namely derrone (**102**), alpinumisoflavone (**103**), auriculasin (**104**), maackianin (**105**) and maackiain (**106**) from the fruits of *L. barbarum* [56,75,76].

#### 2.2.6. Amides **107**–**143**

**Figure 8:**
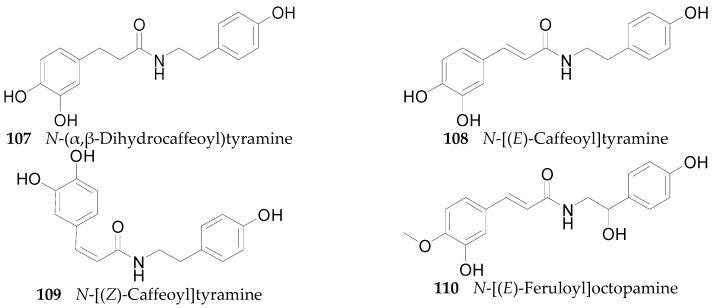

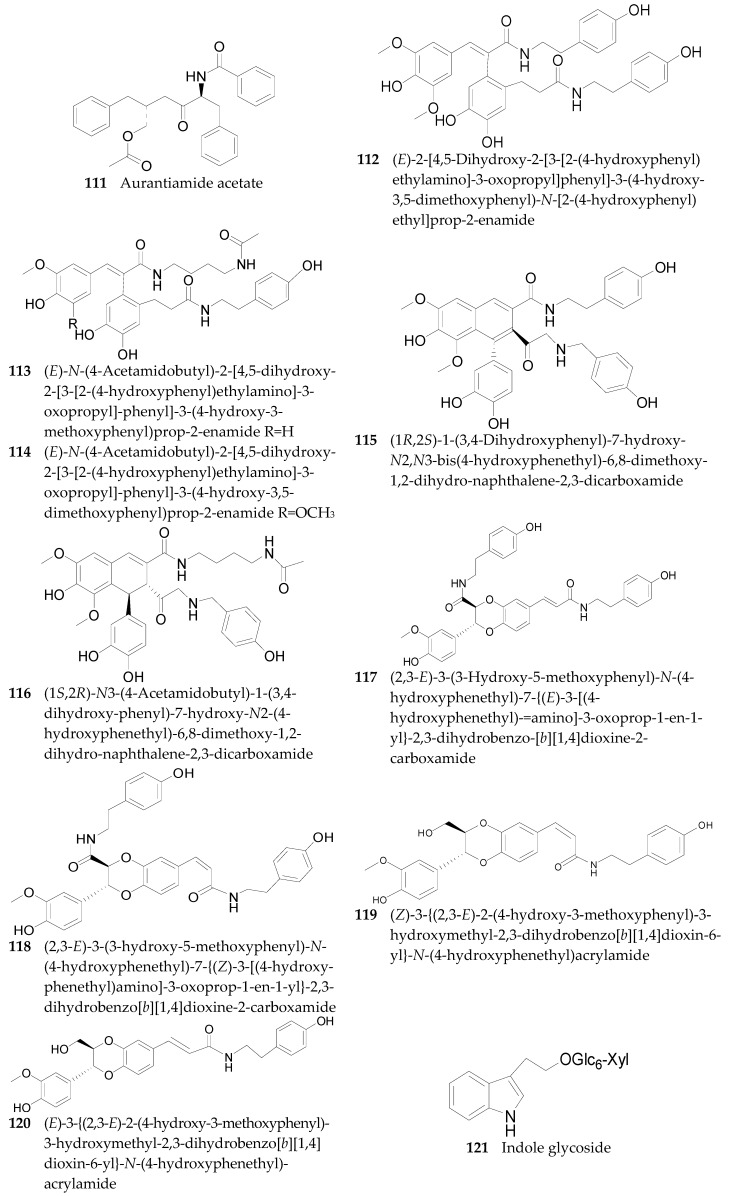

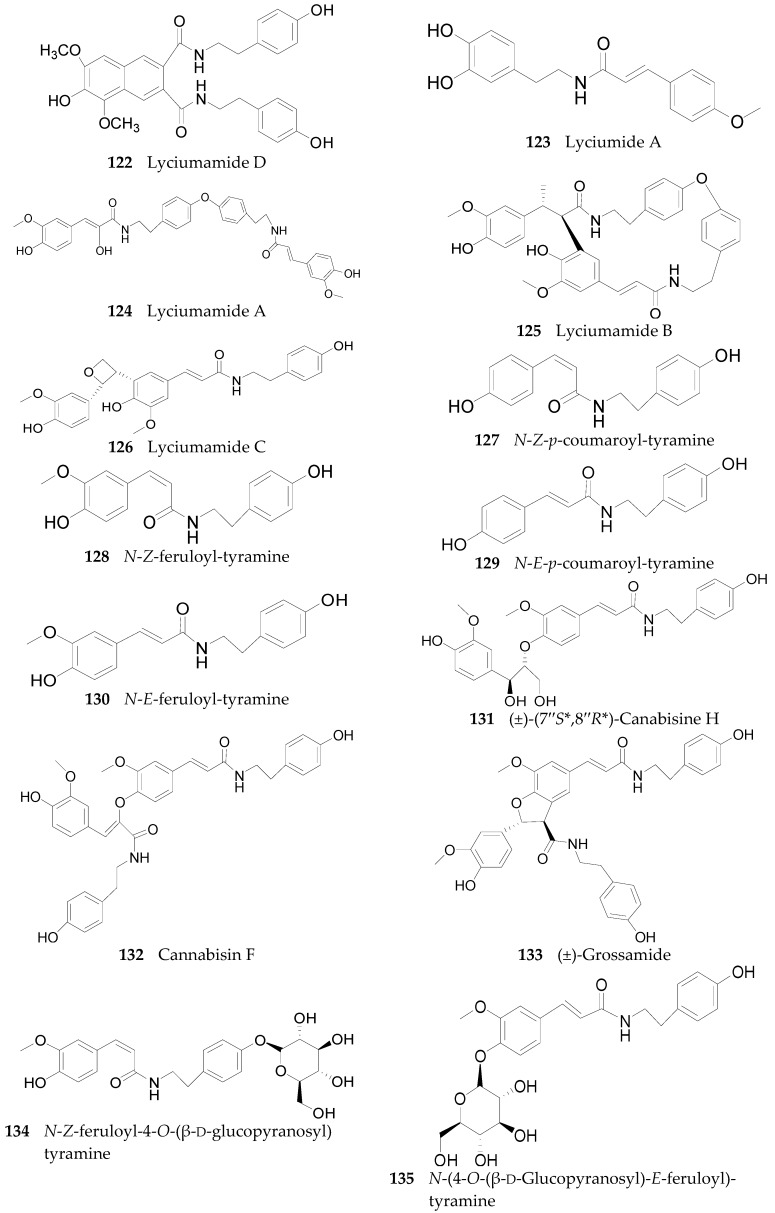

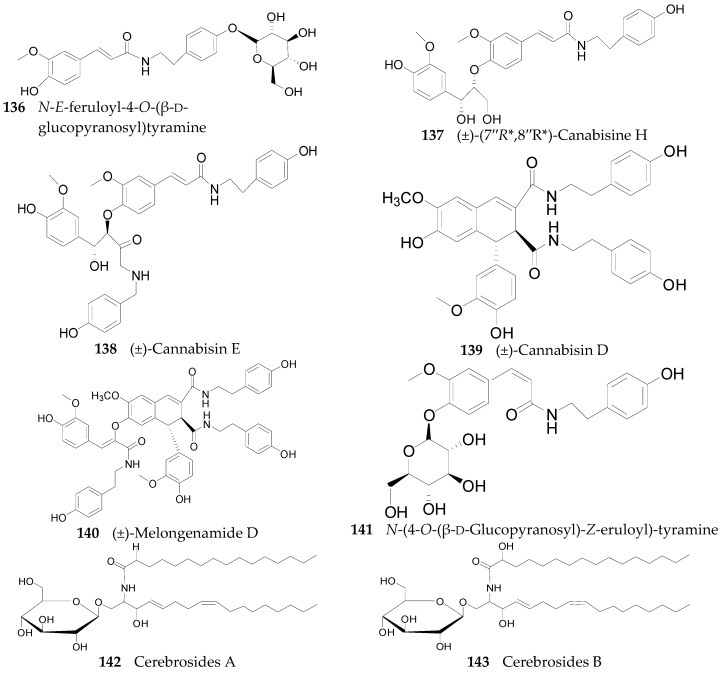
Chemical structures of compounds **107**–**143**.

Sixteen amides **107**–**122** have been isolated from the root bark of *L. chinense* [9,54,60,77,78,79,80], 19 amides (**123**–**141**) have been isolated from the fruits of *L. barbarum* [81,82,83,84,85,86,87,88]. Meanwhile, two cerebrosides **142** and **143** have been obtained from fruits of *L. chinense* [89]. The chemical structures of these amides are shown in Figure 8.

#### 2.2.7. Alkaloids **144**–**215**

To date, 72 alkaloids have been identified, which can be classified into five categories: nortropane, imidazole, piperidine, pyrrole, spermine, tropane, and other alkaloids.

##### Nortropane Alkaloids

**Figure 9:**
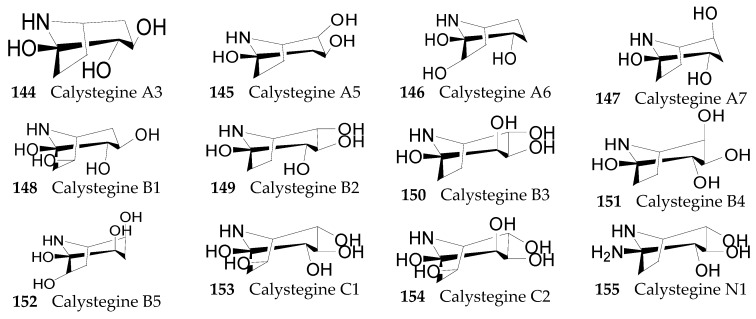

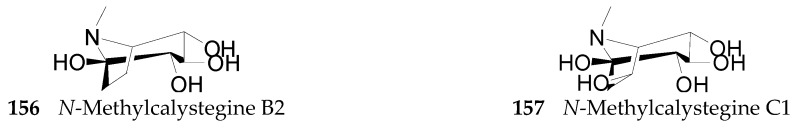
Chemical structures of compounds **144**–**157**.

Fourteen nortropane alkaloids **144**–**157**, shiwn in Figure 9, have been isolated from the root bark of *L. chinense* [90].

##### Imidazole Alkaloids

**Figure 10:**
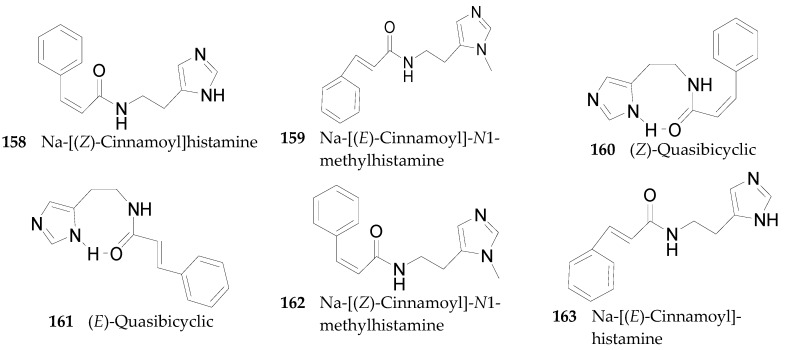
Chemical structures of compounds **158**–**163**.

Six imidazole alkaloids **158**–**162** were detected in the leaves of *L. cestroides* [91]: Meanwhile, one imidazole, Na-[(*E*)-cinnamoyl]histamine (**163**), was obtained from the leaves of *L. barbarum* [66], listed in Figure 10.

##### Piperidine Alkaloids

**Figure 11:**
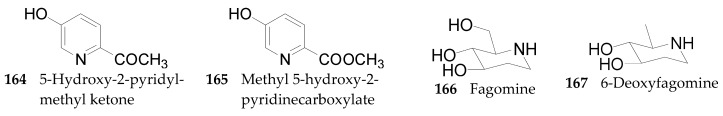
Chemical structures of compounds **164**–**167**.

5-hydroxy-2-pyridylmethyl ketone (**164**), methyl 5-hydroxy-2-pyridinecarboxylate (**165**), fagomine (**166**), and 6-deoxyfagomine (**167**), listed in Figure 11, have been isolated and identified from the genus *Lycium*; among them. Compounds **164** and **165** are from the fruits of *L. barbarum* [92], and **166** and **167** are from the root bark of *L. chinense* [90].

##### Pyrrole Alkaloids

**Figure 12:**
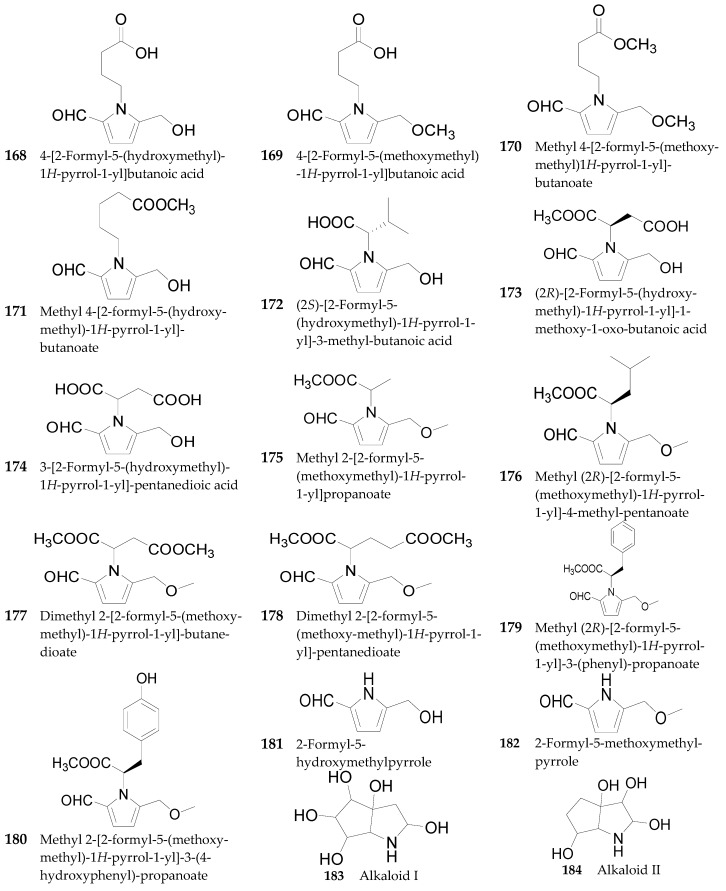
Chemical structures of compounds **168**–**184**.

Thirteen pyrrole alkaloids **168**–**180** have been isolated from the fruits of *L. chinense* [93,94,95]. Likewise, 2-formyl-5-hydroxymethylpyrrole (**181**) and 2-formyl-5-methoxymethylpyrrole (**182**) were isolated from the fruits of *L. barbarum* [92]. Two pyrrolidine alkaloids, alkaloid I (**183**) and alkaloid II (**184**), are obtained from the root bark of *L. chinense* [96]. The chemical structures of these pyrrole alkaloids are listed in Figure 12.

##### Spermine Alkaloids

**Figure 13:**
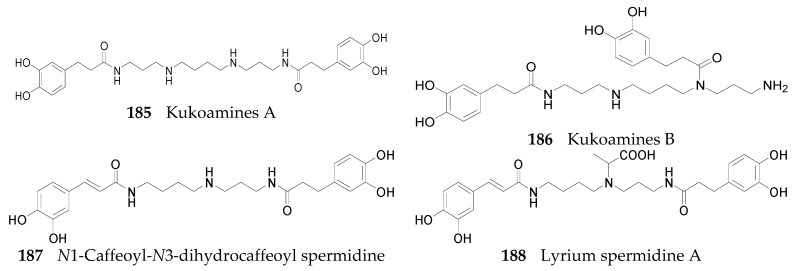
Chemical structures of compounds **185**–**188**.

**Figure 14:**
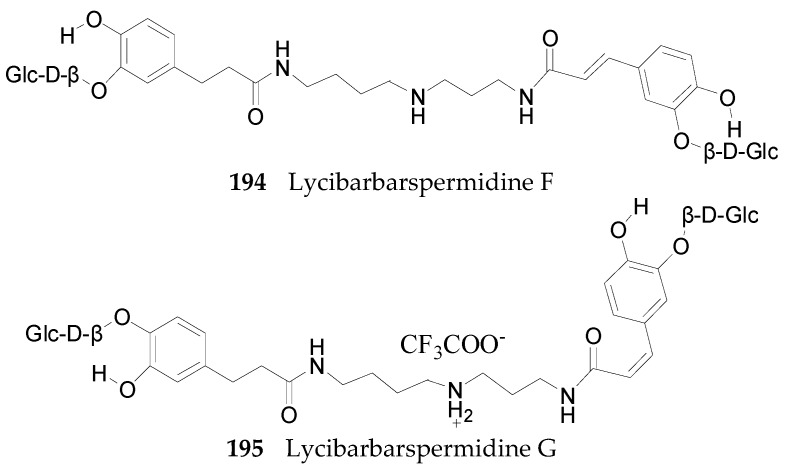
Chemical structures of compounds **194** and **195**.

**Figure 15:**
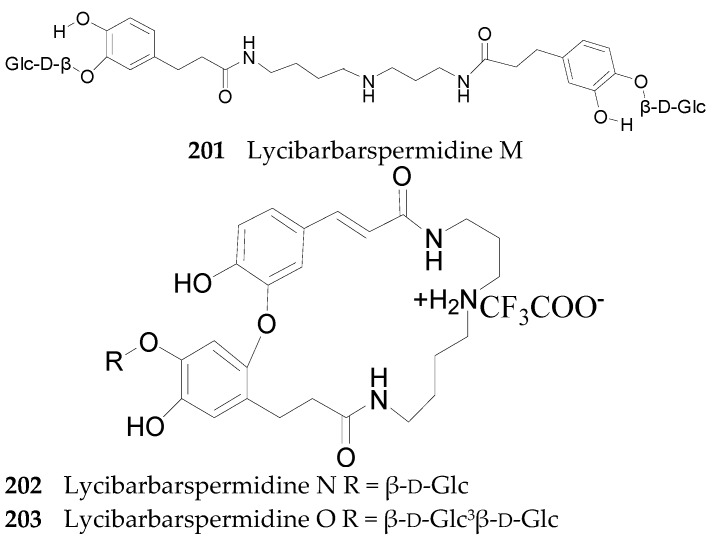
Chemical structures of compounds **201**–**203**.

Nineteen spermine alkaloids have been found in the genus *Lycium*. Kukoamines A (**185**) and kukoamines B (**186**) are from the root bark of *L. chinense* [97,98], while *N*1-caffeoyl-*N*3-dihydrocaffeoyl spermidine (**187**) and lyrium spermidine A (**188**) are from the fruits of *L. ruthenicum* [62,99], listed in Figure 13. Another 15 spermine alkaloids, lycibarbarspermidine A–O (**189**–**203**), listed in Table 9 and Table 10 and Figure 13, Figure 14 and Figure 15, are from *L. barbarum* [100].

##### Tropane Alkaloids

As we know, the genus *Lycium* has been used as both a medicine and a food for a long time in Asia, particularly in China. However, the safety of *Lycium* has been questioned for some time, especially after the detection of the three tropane alkaloids atropine (**204**), hyoscyamine (**205**), and scopolamine (**206**) [101]. Atropine and hyoscyamine were identified from the fruits of *L. barbarum* gathered in India, while scopolamine was identified from *L. halimifolium* at concentrations higher than the toxic dose. However, another scholar, seeking to verify these reports, demonstrated that the atropine content of *L. barbarum* from different sources was just 3.0 ppb—far below the poisoning dose [102]. It was demonstrated that none of the toxic compounds were detected in fruits, leaves, stems and roots of three *L. barbarum* varieties (‘No. 1’, ‘New Big’ and ‘Amber Sweet Goji’) by densitometric TLC analysis [103]. Through field investigation and model specimen inspections, the above three tropane alkaloids were determined to be from *Lycium europaeum* rather than the *L. barbarum*. Thus, the genus *Lycium* is likely non-toxic, and consumers can rest assured that its use is safe [104]. 

**Figure 16:**
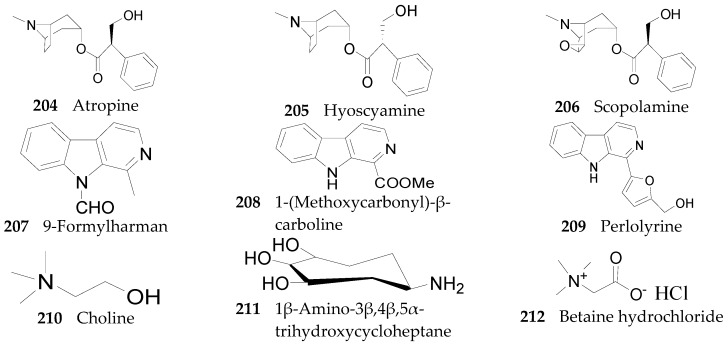

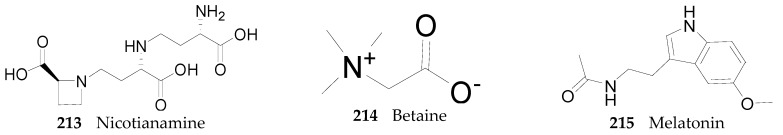
Chemical structures of compounds **204**–**215**.

Other than the alkaloids that have been already mentioned, there are nine others that have been obtained from this genus, including 9-formylharman (**207**), 1-(methoxycarbonyl)-β-carboline (**208**), perlolyrine (**209**), choline (**210**), 1β-amino-3β,4β,5α-trihydroxycycloheptane (**211**), betaine hydrochloride (**212**), nicotianamine (**213**), betaine (**214**), and melatonin (**215**). Compounds **207**–**209** were isolated from the fruits of *L. chinense* [105], while **210**–**212** were isolated from the root bark of *L. chinense* [90]. Compound **213** was isolated from the leaves and flowers of *L. chinense* [106], and **214** and **215** were isolated from the fruits of *L. barbarum* [107,108]. The chemical structures of these tropane alkaloids are listed in Figure 16. 

#### 2.2.8. Anthraquinones **216**–**219**

**Figure 17:**
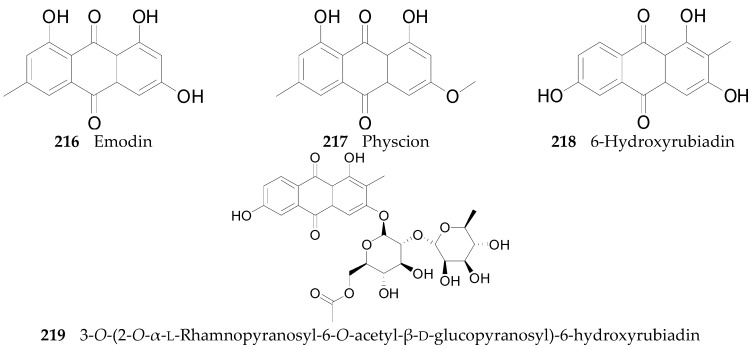
Chemical structures of compounds **216**–**219**.

Four anthraquinones: emodin (**216**), physcion (**217**), 6-hydroxyrubiadin (**218**), and 3-*O*-(2-*O*-α-l-rhamnopyranosyl-6-*O*-acetyl-β-d-glucopyranosyl)-6-hydroxy-rubiadin (**219**), listed in Figure 17, have been obtained from the root bark of *L. chinense* [61,109].

#### 2.2.9. Organic Acids **220**–**251**

**Figure 18:**
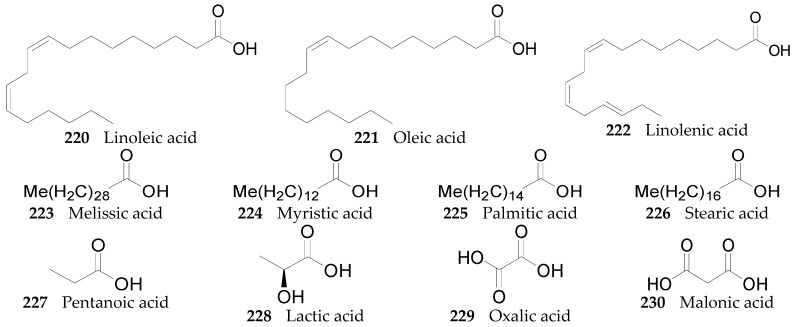

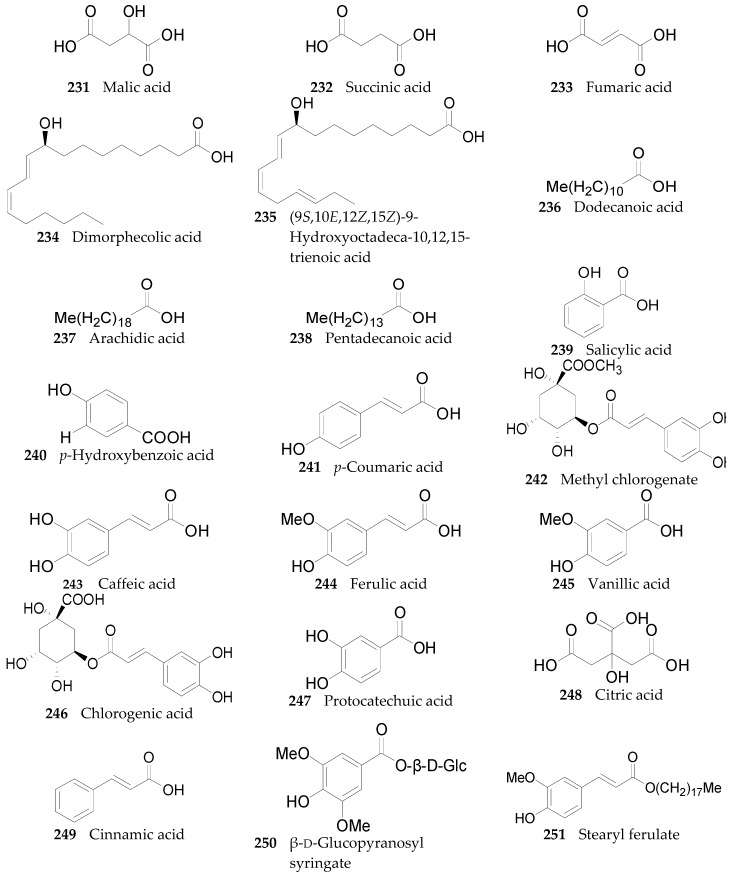
Chemical structures of compounds **220**–**251**.

To this point, 32 organic acids, listed in Figure 18, have been identified from the genus *Lycium*, which can be classified into two groups: aliphatic acids **220**–**238** and aromatic acids and their derivatives **239**–**251**. Compounds **220**–**225** and **240**–**244** were isolated from the fruits of *L. barbarum* [56,63,65,107,110,111,112]; **239** and **245** were isolated from the leaves of *L. barbarum* [66]; **226** was isolated from the root of *L. chinense* [113]; **227**, **248** and **249** were isolated from the fruits of *L. chinense* [70,114]; **228**–**233** and **248** were isolated from the leaves of *L. chinense* [115]; **234**, **235**, and **249**–**251** were isolated from the root bark of *L. chinense* [53,78,93,116,117], and **236**–**238** were isolated from the fruits of *L. urcomanicum* [118,119].

#### 2.2.10. Terpenoids **252**–**290**

**Figure 19:**
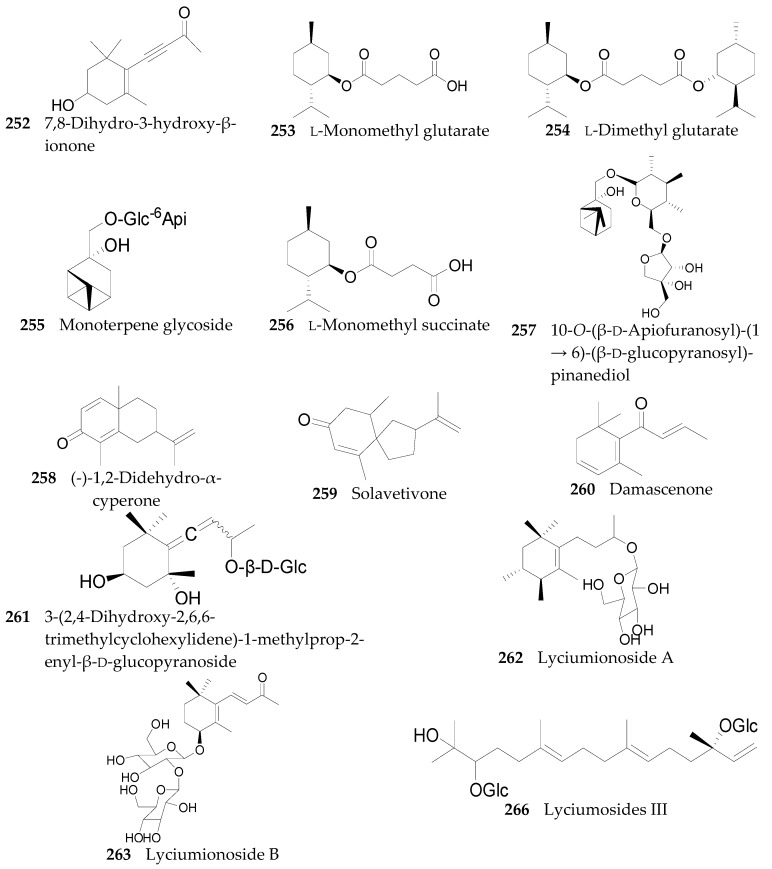
Chemical structures of compounds **252**–**263**, **266**.

**Figure 20:**
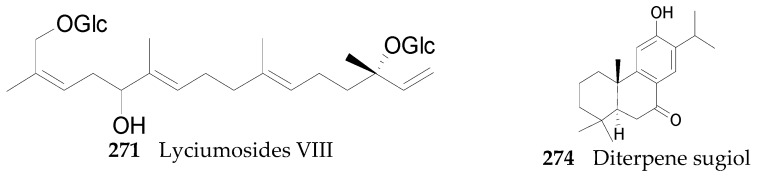
Chemical structures of compounds **271** and **274**.

**Figure 21:**
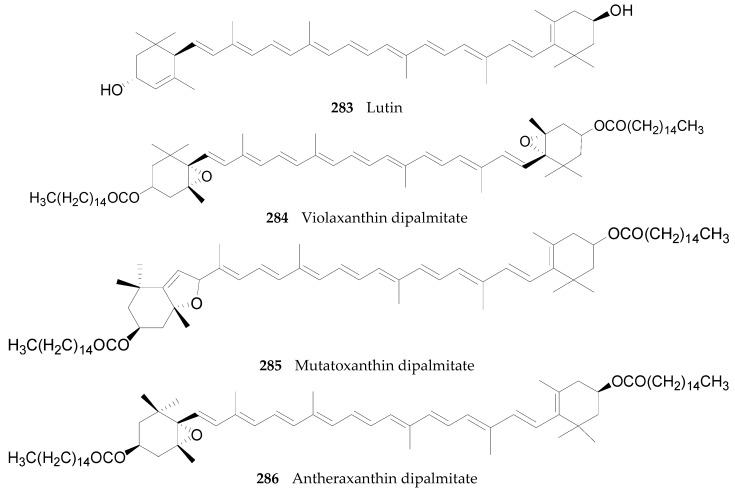

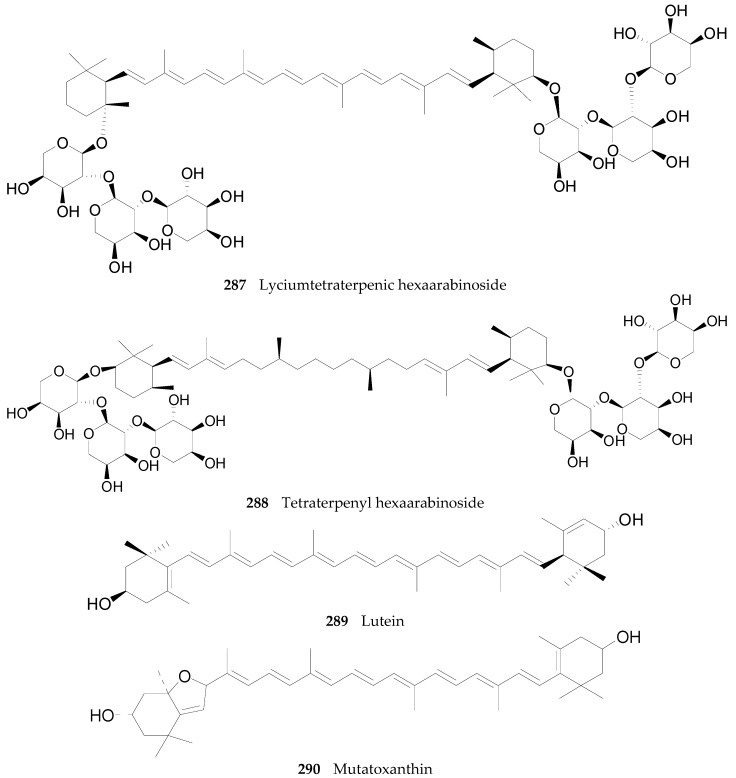
Chemical structures of compounds **283**–**290**.

Thirty-seven terpenoids, listed in Figure 19, Figure 20 and Figure 21 and Table 11 and Table 12, have been found in the genus *Lycium*, mainly including monoterpenes **252**–**256**, sesquiterpenes **257**–**263**, diterpenoids **264**–**274**, and carotenoids **275**–**290**. Among them, carotenoids are one of the more important constituents of the *Lycium* fruits. Thus compounds **256** and **275**–**286** were isolated from the fruits of *L. barbarum* [120,121,122,123]; **253**, **254**, **258**, **259** and **287**–**290** were isolated from the fruits of *L. chinense* [120,121,124,125,126]; **252** and **264**–**272** were isolated from the leaves of *L. chinense* [127,128]; **255**, **258** and **273**–**274** were isolated from the root bark of *L. chinense* [80,116]; **260** and **261** were isolated from the leaves of *L. halimifolium* [23]; and **262** and **265** were isolated from the leaves of *L. barbarum* [129].

#### 2.2.11. Sterols, Steroids, and Their Derivatives **291**–**347**

**Figure 22:**
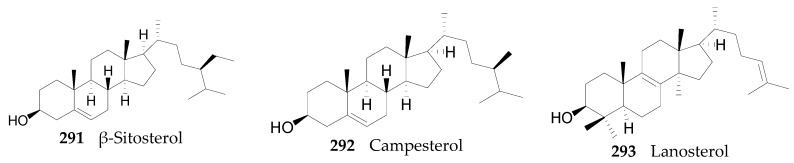

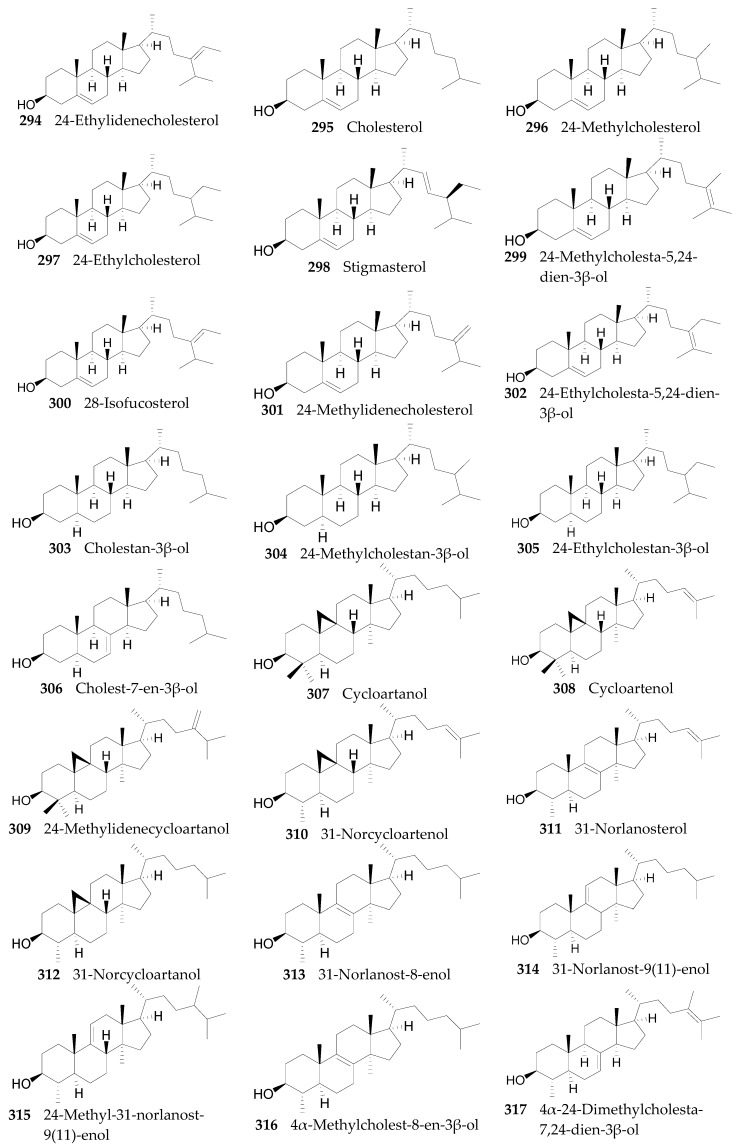

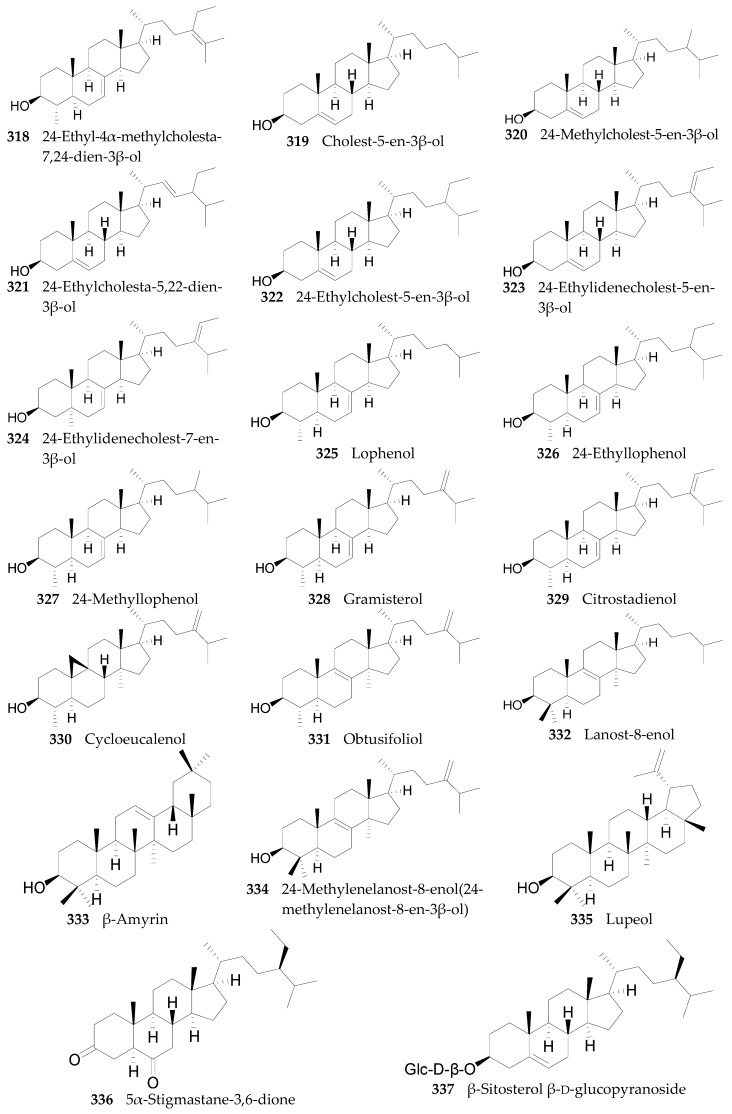

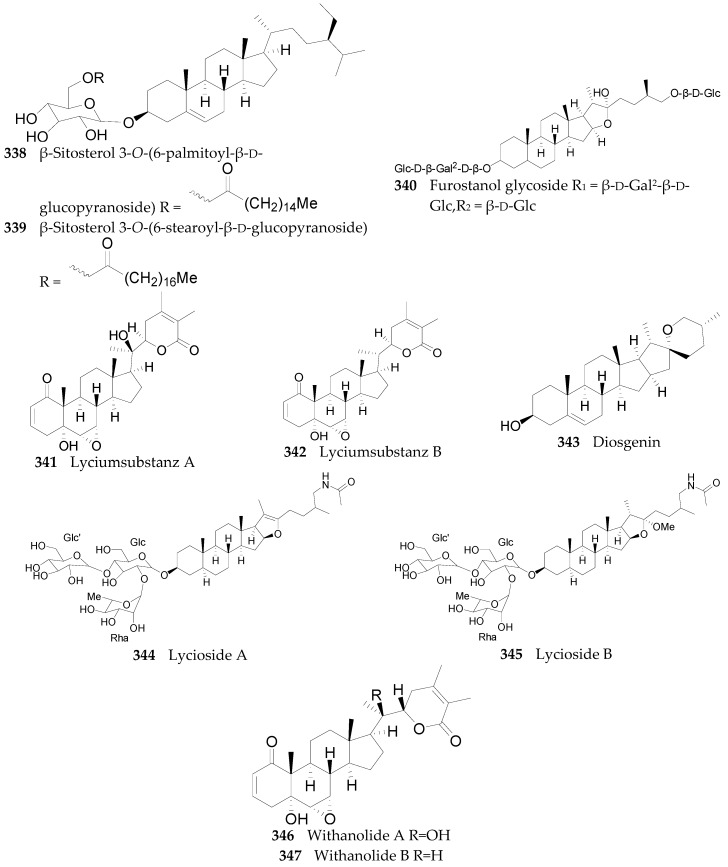
Chemical structures of compounds **291**–**347**.

Fifty-seven sterols, steroids, and their derivatives **291**–**347**, listed in Figure 22, have been identified from the genus *Lycium*, mainly from the seeds and the fruits. Compounds **293** and **343** were identified from the flowers of *L. barbarum* [130], **291**–**292**; **295**, **298**, **319**–**324** and **337**–**339** were identified from the fruits of *L. chinense* [23,35,52,63,107,131]; **341 342**, **346** and **347** were identified from the leaves of *L. chinense* [132,133]; **336** and **340** were identified from the root bark of *L. chinense* [80,121]; **294** was identified from the seed of *L. ciliatum* [66]; all others were identified from the seed of *L. chinense* [134,135,136,137] **344** and **345** were identified from the seeds of *L. barbarum* [138].

#### 2.2.12. Peptides **348**–**352**

**Figure 23:**
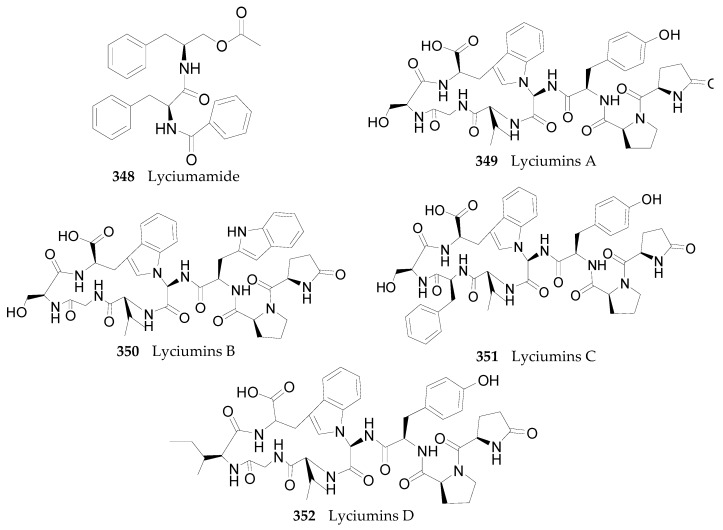
Chemical structures of compounds **348**–**352**.

Five peptides have been isolated from the root bark of *L. chinense* [80,139], including one dipeptide, lyciumamide (**348**), and four octapeptides, called lyciumins A–D (compounds **349**–**350**), illustrated in Figure 23.

#### 2.2.13. Other Compounds **353**–**355**

**Figure 24:**
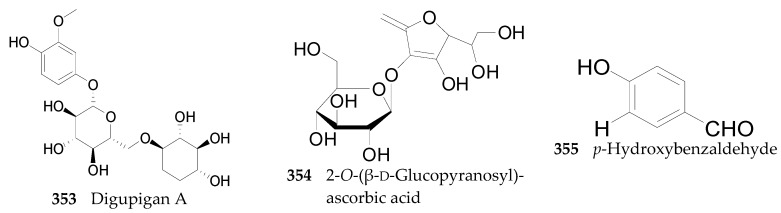
Chemical structures of compounds **353**–**355**.

Other than what has already been mentioned, a few other chemical constituents, listed in Figure 24, were also isolated from the genus *Lycium*. Digupigan A (**353**), 2-*O*-(β-d-glucopyranosyl)ascorbic acid (**354**) and *p*-hydroxybenzaldehyde (**355**) also have been obtained from the root bark of *L. chinense*, the fruits of *L. chinense,* and the fruits of *L. barbarum* [75,76,121,137,140,141], respectively. Many minerals, amino acids, and proteins have also been found in the genus *Lycium,* such as Ca, Mg, Zn, Fe, aminoethanesulfonic acid, γ-aminobutyric acid (GABA), Mn-SOD, etc. [121,142,143].

## 3. Discussion

*Lycium* species are of valuable medicinal, nutritional and functional significance, and have been studied in terms of their chemical compounds. Phytochemical investigations on eight different species, have resulted in the isolation of at least **355** constituents up to July of 2016. Research on chemical compounds has concentrated mainly on *L. barbarum* and *L. chinense*. Therefore, future phytochemistry research should be focused on the other species in *Lycium* genus. In addition, diverse plant parts (i.e., the flowers, leaves, seeds) have also been testified to contain new constituents, most of which possess the novel chemical structures. Polysaccharides play a particularly significant role in exerting pharmacological actions. A specific class of polysaccharides, abbreviated as LBP, is used as biomarker in the 2015 Chinese Pharmacopoeia as a measure by which wolfberry is qualified. At present, LBP in products or in pharmacological studies usually are polysaccharide mixtures with heterogeneity and polydispersity. On the other hand, development of new separation, detection techniques will greatly benefit the phytochemical isolation and structural elucidation of LBP. There is a growing recognition that not only the LBP, but also the plant secondary metabolites may have the potential active ingredients, while most of the research on goji berry was LBP rather than small molecule substances, so more intensive studies of goji berry are required to shed some light on these compounds.

## Figures and Tables

**Table 1:** The molar ratios and source of LBPs.

LBPs	Molar Ratio	Source	Reference
LbGp1	Ara:Gal:Glc = 2.5:1.0:1.0	*L. barbarum*	[26]
LbGp2	Ara:Gal = 4:5	*L. barbarum*	[27]
LbGp3	Ara:Gal = 1:1	*L. barbarum*	[28,29]
LbGp4	Ara:Gal:Rha:Glc = 1.5:2.5:0.43:0.23	*L. barbarum*	[28,30]
LbGp5	Rha:Ara:Xyl:Gal:Man:Glc = 0.33:0.52:0.42:0.94:0.85:1	*L. barbarum*	[28]
LbGp5B	Rha:Ara:Glc:Gal = 0.1:1:1.2:0.3	*L. barbarum*	[31]
LBP3p	Rha:Ara:Xyl:Gal:Man:Glc = 1.25:1.10:1.76:1:1.95:2.12	*L. barbarum*	[32]
LBPC_2_	Xyl:Rha:Man = 8.8:2.3:1	*L. barbarum*	[33]
LBPC_4_	Glc	*L. barbarum*	[33]
LBPA1	heteroglycan	*L. barbarum*	[33]
LBPA3	heteroglycan	*L. barbarum*	[33]
LBP1a-1	Glc	*L. barbarum*	[34]
LBP1a-2	Glc	*L. barbarum*	[34]
LBP3a-1	GalA	*L. barbarum*	[34]
LBP3a-2	GalA	*L. barbarum*	[34]
LBPF1	-	*L. barbarum*	[35]
LBPF2	-	*L. barbarum*	[35]
LBPF3	-	*L. barbarum*	[35]
LBPF4	-	*L. barbarum*	[35]
LBPF5	Ara, Man, Xyl, Glu, Rha	*L. barbarum*	[35,36]
LBPF6	-	*L. barbarum*	[36]
LPBC4	Glc	*L. barbarum*	[37]
LBP-1	Rha:Ara:Xyl:Gal:Man:GalA = 1:7.85:0.37:0.65:3.01:8.16	*L. barbarum*	[22]
WSP1	Rha:Fuc:Ara:Xyl:Man:Gal:Glc = 1.6:0.2:51.4:4.8:1.2:25.9:7.3	*L. barbarum*	[23]
AGP	Rha:Ara:Xyl:Gal:Glc:GalA:GlcA = 3.3:42.9:0.3:44.3:2.4:7.0	*L. barbarum*	[38]
LBP-IV	Rha:Ara:Xyl:Glc:Gal = 1.61:3.82:3.44:7.54:1.00	*L. barbarum*	[39]
LbGp1	Ara:Gal = 5.6:1	*L. barbarum*	[40]
LBP-s-1	Rha:Ara:Xyl:Man:Glu:Gal:Gal A = 1.00:8.34:1.25:1.26:1.91:7.05:15.28	*L. barbarum*	[41]
p-LBP	Fuc:Rha:Ara:Gal:Glc:Xyl:Gal A:Glc A = 1.00:6.44:54.84:22.98:4.05:2.95:136.98:3.35	*L. barbarum*	[42]
Cp-2-A	Ara:Gal:Man:Rha:Glu = 6.02:2.71:1.00:0.70:0.67	*L. chinese*	[43,44]
Cp-2-B	Ara:Gal = 1:0.96	*L. chinese*	[43,44]
Hp-2-A	Ara:Gal = 5.2:1	*L. chinese*	[43,44]
Hp-2-B	Ara:Gal = 7.9:1	*L. chinese*	[43,44]
Hp-2-C	Ara:Gal = 1.2:1	*L. chinese*	[43,44]
Hp-0-A	Ara:Gal = 14:1	*L. chinese*	[43,44]
Cp-1-A	Ara:Xyl = 1:1	*L. chinese*	[45]
Cp-1-B	Ara	*L. chinese*	[45]
Cp-1-C	Ara:Gal = 3:1	*L. chinese*	[45]
Cp-1-D	Ara:Gal = 1:1	*L. chinese*	[45]
LRGP1	Rha:Ara:Xyl:Man:Glu:Gal = 0.65:10.71:0.33:0.67:1:10.41	*L. ruthenicum*	[46]
LRGP2	-	*L. ruthenicum*	[47]
LRGP3	Rha:Ara:Gal = 1.0:14.9:10.4	*L. ruthenicum*	[48]
LRGP4-A	Rha:Ara:Glu:Gal = 1:7.6:0.5:8.6	*L. ruthenicum*	[49]
LRGP5	Rha:Ara:Xyl:Gal:GalA = 1.0:2.2:0.5:1.2:4.7	*L. ruthenicum*	[50]
LRLP4-A	Rha:Ara:Gal = 1:10.3:5.3	*L. ruthenicum*	[47]
LBLP5-A	-	*L. ruthenicum*	[51]

**Table 2:** Chemical structures of compounds **1**–**17**.

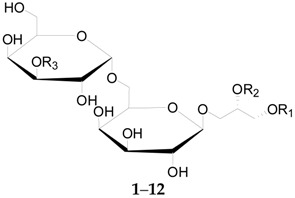			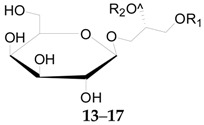		
No.	Compounds	R_1_	R_2_	R_3_	Source
**1**	Glycerogalactolipids A	Palmitoyl	Linolenoyl	Linolenoyl	*L. barbarum*
**2**	Glycerogalactolipids B	Palmitoyl	Linolenoyl	Linoleoyl	*L. barbarum*
**3**	Glycerogalactolipids C	Palmitoyl	Linolenoyl	Palmitoyl	*L. barbarum*
**4**	Glycerogalactolipids D	Palmitoyl	Linoleoyl	Palmitoyl	*L. barbarum*
**5**	Glycerogalactolipids E	Palmitoyl	Palmitoyl	Palmitoyl	*L. barbarum*
**6**	Glycerogalactolipids F	Palmitoyl	Palmitoyl	H	*L. barbarum*
**7**	Glycerogalactolipids G	Linolenoyl	Linolenoyl	H	*L. barbarum*
**8**	Glycerogalactolipids H	Linolenoyl	Linoleoyl	H	*L. barbarum*
**9**	Glycerogalactolipids I	Palmitoyl	Linolenoyl	H	*L. barbarum*
**10**	Glycerogalactolipids J	Palmitoyl	Linoleoyl	H	*L. barbarum*
**11**	Glycerogalactolipids K	Palmitoyl	Oleoyl	H	*L. barbarum*
**12**	Glycerogalactolipids L	Stearoyl	Linoleoyl	H	*L. barbarum*
**13**	Glycerogalactolipids M	Palmitoyl	Linolenoyl	–	*L. barbarum*
**14**	Glycerogalactolipids N	Palmitoyl	Linoleoyl	–	*L. barbarum*
**15**	Glycerogalactolipids O	Palmitoyl	Oleoyl	–	*L. barbarum*
**16**	Glycerogalactolipids P	Linolenoyl	Linolenoyl	–	*L. chinense*
**17**	Glycerogalactolipids Q	Linoleoyl	Linolenoyl	–	*L. chinense*

**Table 3:** Chemical structures of compounds **26**–**28**.

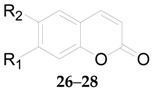				
No.	Compounds	R_1_(R)	R_2_	Source
**26**	Isoscopoletin	OCH_3_	OH	*L. barbarum*
**27**	Scopolin	*O*-β-d-Glc	OCH_3_	*L. chinense*
**28**	Fabiatrin	*O*-β-d-Glc^6^-β-d-Xyl	OCH_3_	*L. chinense*

**Table 4:** Chemical structures of compounds **32**–**51**.

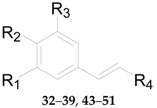			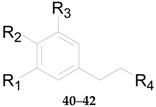			
No.	Compounds	R_1_	R_2_	R_3_	R_4_	Source
**32**	1-*O*-*E*-feruloyl-6-*O*-β-d-xylopyranosyl-β-d-glucopyranoside	OCH_3_	OH	H	COO-β-d-Glc^6^-β-d-Xyl	*L. barbarum*
**33**	6-*O*-*E*-feruloyl-2-*O*-β-d-glucopyranosyl-α-d-glucopyranoside	OCH_3_	OH	H	COO^6^-*α*-d-Glc^2^-β-d-Glc	*L. barbarum*
**34**	1-*O*-*E*-feruloyl-β-d-glucopyranoside	OCH_3_	OH	H	COO-β-d-Glc	*L. barbarum*
**35**	Ethyl-4-*O*-β-d-glucopyranosyl-*E*-ferulate	OCH_3_	*O*-β-d-Glc	H	COOCH_2_CH_3_	*L. barbarum*
**36**	Ethyl *E*-ferulate	OCH_3_	OH	H	COOCH_2_CH_3_	*L. barbarum*
**37**	*E*-sinapinic acid	OCH_3_	OH	OCH_3_	COOH	*L. barbarum*
**38**	Syringenin	OCH_3_	OH	OCH_3_	CH_2_OH	*L. barbarum*
**39**	*E*-ferulic acid	OCH_3_	OH	H	COOH	*L. barbarum*
**40**	Phloretic acid	H	OH	H	COOH	*L. barbarum*
**41**	Dihydroferulic acid	OCH_3_	OH	H	COOH	*L. barbarum*
**42**	Ethyl dihydroferulate	OCH_3_	OH	H	COOCH_2_CH_3_	*L. barbarum*
**43**	Lycibarbarphenylpropanoids A	H	OH	H	COO-β-d-Glc^3^-β-d-Glc	*L. barbarum*
**44**	Lycibarbarphenylpropanoids B	H	OH	H	COO-β-d-Glc^4^-β-d-Glc	*L. barbarum*
**45**	Lycibarbarphenylpropanoids C	OCH_3_	OH	H	COO-β-d-Glc^3^-β-d-Glc	*L. barbarum*
**46**	Lycibarbarphenylpropanoids D	OCH_3_	OH	H	COO-β-d-Glc^4^-β-d-Glc	*L. barbarum*
**47**	Lycibarbarphenylpropanoids E	OCH_3_	OH	H	CH_2_O-β-d-Glc^3^-β-d-Glc	*L. barbarum*
**48**	Lycibarbarphenylpropanoids F	H	*O*-β-d-Glc^3^-β-d-Glc	H	COOCH_2_CH_3_	*L. barbarum*
**49**	Lycibarbarphenylpropanoids G	H	*O*-β-d-Glc^4^-β-d-Glc	H	COOCH_2_CH_3_	*L. barbarum*
**50**	Lycibarbarphenylpropanoids H	OCH_3_	*O*-β-d-Glc^4^-β-d-Glc	H	COOCH_2_CH_3_	*L. barbarum*
**51**	Lycibarbarphenylpropanoids I	*O*-β-d-Glc	OH	H	COOCH_2_CH_3_	*L. barbarum*

**Table 5:** Chemical structures of compounds **58**–**60**.

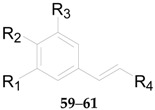						
No.	Compounds	R_1_	R_2_	R_3_	R_4_	Source
**58**	6-*O*-*E*-*p*-coumaroyl-2-*O*-β-d-glucopyranosyl-α-d-glucopyranoside	H	OH	H	COO^6^-*α*-d-Glc^2^-β-d-Glc	*L. barbarum*
**59**	Ethyl-4-*O*-β-d-glucopyranosyl-*E*-*p*-coumarate	H	*O*-β-d-Glc	H	COOCH_2_CH_3_	*L. barbarum*
**60**	Ethyl *E*-*p*-coumarate	H	OH	H	COOCH_2_CH_3_	*L. barbarum*

**Table 6:** Chemical structures of compounds **62** and **65**–**67**.

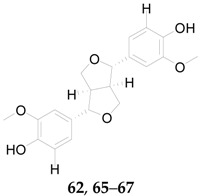					
No.	Compounds	R_1_	R_2_	R_3_	Source
**62**	Pinoresinol	H	OH	H	*L. barbarum*
**65**	Medioresinol	H	OH	OCH_3_	*L. barbarum*
**66**	Syringaresinol	OCH_3_	OH	OCH_3_	*L. barbarum*
**67**	4-*O*-(β-d-glucopyranosyl)syringaresinol	OCH_3_	*O*-β-d-Glc	OCH_3_	*L. barbarum*

**Table 7:** Chemical structures of compounds **75**–**80**, **82**–**83**, **85**–**87** and **89**–**93**.

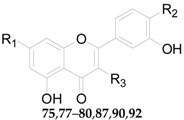		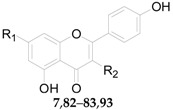		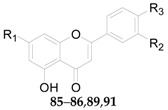	
No.	Compounds	R_1_	R_2_	R_3_	Source
**75**	Quercitrin	OH	OH	O-α-l-Rha	*L. barbarum*
**76**	Kaempferol	OH	OH	–	*L. barbarum*
**77**	Quercetin	OH	OH	OH	*L. barbarum*
**78**	Rutin	OH	OH	*O*-β-d-Glc^6^-α-l-Rha	*L. barbarum*
**79**	Narcissoside	OH	OCH_3_	*O*-β-d-Glc^6^-α-l-Rha	*L. barbarum*
**80**	7-*O*-(β-d-Glucopyranosyl)-rutin	*O*-β-d-Glc	OH	*O*-β-d-Glc^6^-α-l-Rha	*L. barbarum*
**82**	7-*O*-(β-d-Glucopyranosyl)-nicotiflorin	*O*-β-d-Glc	*O*-β-d-Glc^6^-α-l-Rha	–	*L. barbarum*
**83**	7-*O*-(β-d-Glucopyranosyl)-3-*O*-[β-d-glucopyranosyl]-(1 → 2)-β-d-galactop	*O*-β-d-Glc	*O*-β-d-Glc^6^-α-l-Glc	–	*L. barbarum*
**85**	Luteolin	OH	OH	OH	*L. chinense*
**86**	Acacetin	OH	H	OCH_3_	*L. chinense*
**87**	7-*O*-(β-d-Glucopyranosyl)-3-*O*-[β-d-glucopyranosyl-(1 → 2)-β-d-galactopyranosyl]-quercetin	*O*-β-d-Glc	OH	*O*-β-d-Glc^2^-β-d-Glc	*L. chinense*
**89**	7-*O*-[α-l-Rhamno-pyranosyl-(1 → 6)-β-d-glucopyranosyl]-acacetin	*O*-β-d-Glc^6^-α-l-Rha	H	OCH_3_	*L. chinense*
**90**	3-*O*-Sophoroside-quercetin	OH	OH	*O*-β-d-Glc^2^-β-d-Glc	*L. chinense*
**91**	Apigenin	OH	H	OH	*L. chinense*
**92**	Isoquercitrin	OH	OH	*O*-β-d-Glc	*L. halimifolium*
**93**	Nicotiflorin	OH	*O*-β-d-Glc^6^-α-l-Rha	–	*L. halimifolium*

**Table 8:** Chemical structures of compounds **95**–**98**.

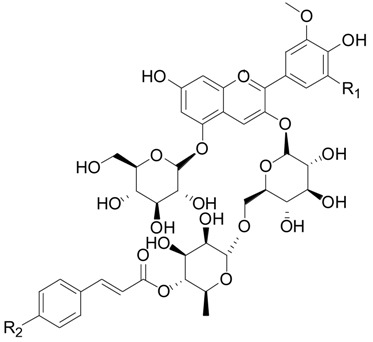				
No.	Compounds	R_1_	R_2_	Source
**95**	5-*O*-(β-d-Glucopyranosyl)-3-*O*-[4-*O*-*p*-*E*-coumaroyl-α-l-rhamnopyranosyl-(1 → 6)-β-d-glucopyranosyl]-peonidin	H	OH	*L. ruthenicum*
**96**	5-*O*-(β-d-Glucopyranosyl)-3-*O*-[4-*O*-*p*-*E*-coumaroyl-α-l-rhamnopyranosyl-(1 → 6)-β-d-glucopyranosyl]-petunidin	OH	OH	*L. ruthenicum*
**97**	5-*O*-(β-d-Glucopyranosyl)-3-*O*-[4-*O*-*p*-*Z*-coumaroyl-α-l-rhamnopyranosyl-(1 → 6)-β-d-glucopyranosyl]-malvidin	OCH_3_	OH	*L. ruthenicum*
**98**	5-*O*-(β-d-Glucopyranosyl)-3-*O*-[4-*O*-*p*-*E*-(β-d-glucopyranoside)-coumaroyl-α-l-rhamnopyranosyl-(1 → 6)-β-d-glucopyranosyl]-petunidin	OH	*O*-β-d-Glc	*L. ruthenicum*

**Table 9:** Chemical structures of compounds **189**–**193**.

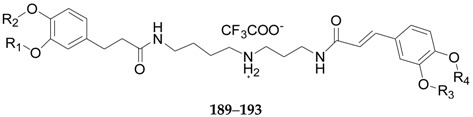						
No.	Compounds	R_1_	R_2_	R_3_	R_4_	Source
**189**	Lycibarbarspermidine A	H	β-d-Glc	H	H	*L. barbarum*
**190**	Lycibarbarspermidine B	H	H	β-d-Glc	H	*L. barbarum*
**191**	Lycibarbarspermidine C	β-d-Glc	H	H	H	*L. barbarum*
**192**	Lycibarbarspermidine D	H	H	H	β-d-Glc	*L. barbarum*
**193**	Lycibarbarspermidine E	H	β-d-Glc	β-d-Glc	H	*L. barbarum*

**Table 10:** Chemical structures of compounds **196**–**200**.

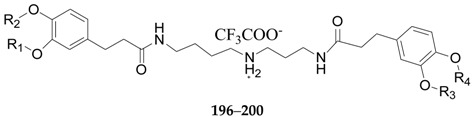						
No.	Compounds	R_1_	R_2_	R_3_	R_4_	Source
**196**	Lycibarbarspermidine H	H	H	H	β-d-Glc	*L. barbarum*
**197**	Lycibarbarspermidine I	H	β-d-Glc	H	H	*L. barbarum*
**198**	Lycibarbarspermidine J	H	*H*	β-d-Glc	H	*L. barbarum*
**199**	Lycibarbarspermidine K	β-d-Glc	*H*	β-d-Glc	H	*L. barbarum*
**200**	Lycibarbarspermidine L	H	β-d-Glc	H	β-d-Glc	*L. barbarum*

**Table 11:** Chemical structures of compounds **264**–**265**, **267**–**270** and **272**–**273**.

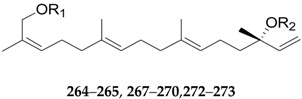				
No.	Compounds	R_1_	R_2_	Source
**264**	Lyciumosides I	Glc	Glc	*L. chinense*
**265**	Lyciumosides II	Glc^2^-Glc	Glc	*L. chinense*
**267**	Lyciumosides IV	Glc	Glc^4^-Rha	*L. chinense*
**268**	Lyciumosides V	Glc^6^-Rha	Glc	*L. chinense*
**269**	Lyciumosides VI	Glc^6^-Rha	Glc^4^-Rha	*L. chinense*
**270**	Lyciumosides VII	Glc^2^-Rha(^6^-Glc)	Glc	*L. chinense*
**272**	Lyciumosides IX	Glc	6-*O*-malonyl-Glc	*L. chinense*
**273**	Capsianoside II	Rha^3^-Glc^6^-Rha	Glc^2^-Glc	*L. chinense*

**Table 12:** Chemical structures of compounds **275**–**282**.

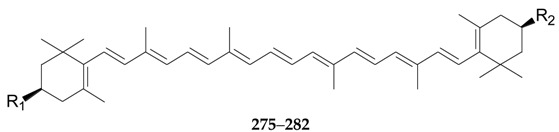				
No.	Compounds	R_1_	R_2_	Source
**275**	β-Carotene	H	H	*L. barbarum*
**276**	β-Cryptoxanthin	OH	H	*L. barbarum*
**277**	Zeaxanthin	OH	OH	*L. barbarum*
**278**	Zeaxanthin monopalmitate	OCO(CH_2_)_14_CH_3_	OH	*L. barbarum*
**279**	Zeaxanthin dipalmitate	OCO(CH_2_)_14_CH_3_	OCO(CH_2_)_14_CH_3_	*L. barbarum*
**280**	Zeaxanthin monomyristate	OH	OCO(CH_2_)_12_CH_3_	*L. barbarum*
**281**	Zeaxanthin dimyristate	OCO(CH_2_)_12_CH_3_	OCO(CH_2_)_12_CH_3_	*L. barbarum*
**282**	β-Cryptoxanthin palmitate	OCO(CH_2_)_14_CH_3_	H	*L. barbarum*
